# A Novel and High-Effective Biosynthesis Pathway of Hesperetin-7-O-Glucoside Based on the Construction of Immobilized Rhamnosidase Reaction Platform

**DOI:** 10.3389/fbioe.2020.00608

**Published:** 2020-06-10

**Authors:** Wenjing Wan, Na Xia, Siming Zhu, Qiang Liu, Youcheng Gao

**Affiliations:** ^1^School of Food Science and Engineering, South China University of Technology, Guangzhou, China; ^2^College of Life and Geographic Sciences, Kashi University, Kashi, China

**Keywords:** biosynthesis, immobilized rhamnosidase, Fe_3_O_4_@graphene oxide, hesperetin-7-O-glucoside, hesperidin-Cu (II)

## Abstract

Hesperetin-7-O-glucoside (HMG) is a precursor for synthesizing a sweetener named neohesperidin dihydrochalcone, and the coordination toward flavonoids of metal ions tends to increase the water solubility of flavonoids. In order to achieve effective synthesis of HMG, an immobilized enzyme catalysis platform was constructed using an immobilized rhamnosidase on Fe_3_O_4_@graphene oxide (Fe_3_O_4_@GO), a novel reaction pathway based on the platform was designed for preparing a hesperidin complex as a soluble substrate, and ammonium hydroxide as a ligand dissociation agent to obtain HMG. The Fe_3_O_4_@GO was characterized by Fourier transform infrared (FT-IR), X-ray diffraction (XRD), scanning electron microscope (SEM), and thermal methods (TG/DSC) analysis to evaluate the immobilization matrix properties. The enzyme activity in free and immobilized form at different pH and temperature was optimized. The reusability of immobilized enzyme was also determined. In addition, the kinetic parameters (*K*_m_ and *V*_max_) were computed after experiment. Results indicated that rhamnosidase immobilized on Fe_3_O_4_@GO using a green cross-linker of genipin hydrolyzed successfully and selectively the soluble hesperidin-Cu (II) complex into HMG-Cu (II), a permanent magnet helped the separation of immobilized enzyme and hydrolytes, and ammonium hydroxide was an effective ligand dissociation agent of translating HMG-Cu (II) into HMG with high purity determined by ultraviolet-visible (UV-Vis) spectra analysis and time-of-flight mass spectrometry (TOF-MS). As a result, a novel and high-effective biosynthesis pathway of HMG based on a selectively catalytic reaction platform were constructed successfully. The pathway based on the platform has great potential to produce valuable citrus monoglycoside flavonoid HMG, and the designed reaction route are feasible using the hesperidin-Cu (II) complex with good solubility as a reaction substrate and using ammonium water as a dissociation agent.

## Introduction

Flavanones belong to a unique class of polyphenols containing three main aglycones: hesperetin, eriodictyol and naringenin (Manach et al., 2003). These glycosylated flavonoids are important components of citrus and play a vital role in human health (Carceller et al., 2019). Hesperidin, has been identified as the most abundant flavonoid in citrus peel, followed by naringin, a derivative of hesperidin (Nogata et al., 2006). Some studies have found that hesperidin possesses anti-inflammatory, lipid-lowering, antioxidant and anticancer biological and pharmacological properties (Bok et al., 1999; Choi et al., 2001). Moreover, it can also form metal complexes through the coordination of carbonyl and hydroxyl groups with metal ions to enhance water solubility and bioavailability (Ioannou et al., 2018). Under the action of rhamnosidase, the main hydrolyzed products were monoglycoside flavonoids named HMG which possesses a variety of pharmacological and biochemical activities, as well as anti-inflammatory, antiviral, and anticancer effects (Grazul and Budzisz, 2009). HMG is prepared mainly by chemical hydrolysis, but the reaction conditions are difficult to control. During the acid hydrolysis process, hesperidin is easy to over-hydrolyze the target product HMG to produce hesperetin, resulting in a low HMG yield and environmental pollution. Therefore, the preparation of HMG by chemical method is not suitable for industrial production, while the enzymatic method has the advantages of high specificity and green production. Hydrolyzing hesperidin using rhamnosidase can also obtain HMG, however, the poor solubility of hesperidin limits HMG yield. Perhaps soluble hesperidin-Cu (II) is a potential substitute substrate for hesperidin according to our former study.

Alpha-L-rhamnosidase [EC 3.2.1.40] is an enzyme that exclusively severs the terminal α-L-rhamnose from numerous rhamnosides and has been noted to be widely distributed in nature (Cui et al., 2007). It is not among the most studied glycosidase, but an important biotechnology enzyme. The hydrolysis of rhamnoside has crucial application values in debittering grapefruit juice and other citrus juices (Birgisson et al., 2007; Soria et al., 2012). Besides, there are other applications in the following: enhancing the wine aroma after hydrolysis of terpene glycoside precursors (Manzanares et al., 2003), using as an agent for structural study of glycosides to hydrolyzes L-rhamnosides specifically (Monti et al., 2004), and preparation of raw materials to synthesize several compounds of prospect in the food industry by hydrolysis of natural glycosides (Carceller et al., 2019).

However, free enzyme systems are not widely accepted in industry due to the long processing time, the difficulty in product recovery and enzyme reuse (Cardelle-Cobas et al., 2016). Immobilized enzymes play an important role in food and pharmaceutical benefited from their low cost and simple fixation process. For cells or enzymes, immobilized biocatalysts perform better than free cells or enzymes. It is generally known that the advantages of immobilized enzymes include the residual recovery of products and substrates, the promoted reusability of catalysts (Park and Chang, 2000), inhibition of reducing product (Kosugi and Suzuki, 1992). Due to unique properties like lager specific surface area and high chemical stability, graphene oxide (GO) as a precursor of graphene, has been widely applied in biotechnology and industry (Lomeda et al., 2008; Zhang et al., 2012; Zhao et al., 2014), such as GO has become a suitable carrier material for various enzyme immobilization that considered to be an appropriate method to increase stability and facilitate reuse, and these properties are why they have been used as nanocatalysts in other studies (Maleki and Paydar, 2015; Maleki and Rahimi, 2018). Certainly, enzyme activity after immobilization leading to a significant loss is still a main problem to solve. Until now a mass of carrier and composite materials have been tested for immobilized enzyme (Cheng et al., 2012; Wei et al., 2012). Using magnetic carrier can remarkably improve product enzymes and other components in the reaction mixture degrees of separation versus the pure carrier or non-magnetic materials (Demirel et al., 2004; Jiang et al., 2012). Zhang et al. (2020) considered magnetic nanoparticles to be a significant approach for targeted drug delivery. And magnetic nanocatalysts have also been reported because they are easily separated from the reaction mixture (Maleki et al., 2019). As a result, the preparation of magnetic immobilized enzyme materials has research significance and application value.

Ammonium hydroxide is a common complexing agent whose complexing ability exceeds that of hesperidin and its derivatives like HMG. After the complexing of ammonium and metal, HMG changes from complexing state to free state. Based on the characteristics that the complex of the ammonium-metal complex is easily soluble in water, while the solubility of HMG is poor when the solvent is acidic or neutral, it is separated from the complexing agent and metal complex, thus obtained a high-purity HMG extract. However, this novel dissociation approach has seldom been reported in relevant literatures.

**FIGURE 1:**
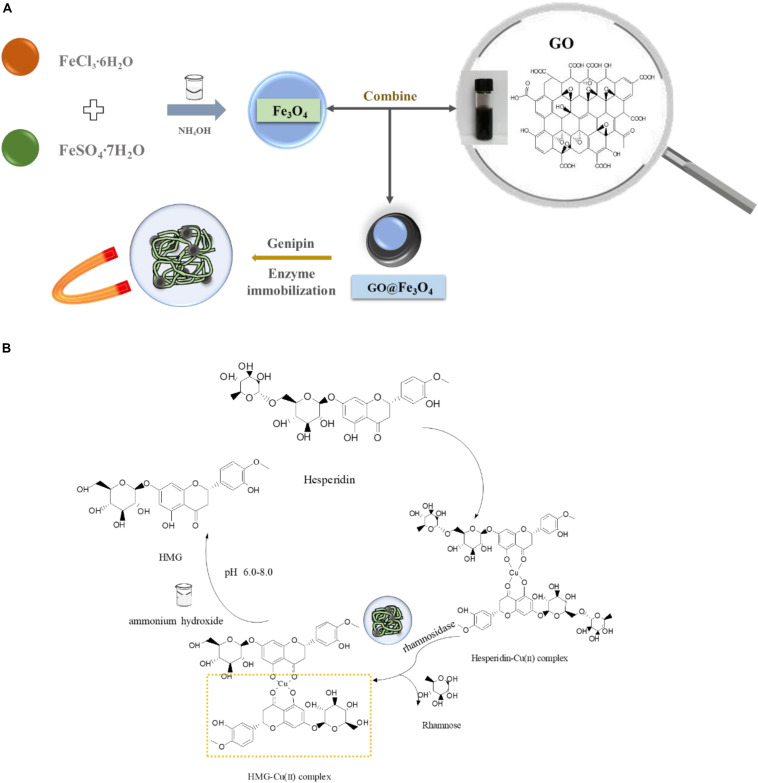
**(A)** Scheme of biosynthesis HMG and **(B)** enzyme-immobilized platform construction based on Fe_3_O_4_@GO composite.

In this paper, a novel and high-effective biosynthesis pathway was developed to produce HMG based on the construction of immobilized rhamnosidase reaction platform using Fe_3_O_4_@GO (Figure 1). This study committed to explore the feasibility and operability of the novel HMG-synthesizing pathway. Synthesis of the Fe_3_O_4_@GO composite materials was the first step for immobilized enzyme, and its structure was characterized by FT-IR, XRD, SEM, and thermal methods analysis. In addition, the enzymatic properties of free enzyme and immobilized enzyme were also tested. Fe_3_O_4_@GO as an immobilized framework support can increase the reuse rate of the enzyme to save resources, and the enzyme activity stability was improved compared to that before immobilization. Moreover, there was a synergistic effect with the product, it can promote the separation of the enzyme and the hydrolysate, to a certain extent, was conducive to the conversion rate of the product. Ammonium hydroxide was used as the ligand dissociation agent to compete with the HMG-copper complex in the enzymatic hydrolysate to combine with copper, so that HMG was extracted and verified by TOF-MS analysis.

## Materials and Methods

### Materials

Graphene oxide was purchased from Zhongsen Leadership Technology Co., Ltd (Shenzhen, China). Ferrous sulfate heptahydrate was obtained from Tianjin Damao Chemical Reagent Factory (Tianjin, China). Iron(III) chloride hexahydrate was purchased by Fuchen Chemical Reagent Co., Ltd (Tianjin, China). Genipin was provided by Liuzhou Qianrong Health Industry Co., Ltd (Liuzhou, Guangxi, China). Hesperidin, rhamnosidase (2.15 U) were provided by Shanghai Yuanye Biotechnology Co., Ltd. (Dongying, Shandong, China). D296 macroporous strong basic anionic exchange resin (referred to as D296 resin) was provided by Zhengzhou Qinshi Technology Co., Ltd. (Zhengzhou, China). Ethanol, copper dichloride, citric acid, and sodium citrate were of analytical grade.

### Preparation and Characterization of Catalyst

#### Synthesis of Fe_3_O_4_@GO

The Fe_3_O_4_@GO was synthesized with a slight modification as described in literature (Kassaee et al., 2011). In the presence of GO, the composite was prepared from ferric chloride and ferrous sulfate heptahydrate at a molar ratio of 2:1. 100 mg of GO in 50 mL of distilled water was ultrasonicated for 30 min, FeCl_3_ and FeSO_4_ (8 × 10^–3^ and 4 × 10^–3^ mol/L, respectively) were added and stirred vigorously at room temperature. After dissolution, the temperature was raised to 80°C and ammonium hydroxide was dropped into the solution to raise the solution pH to 10.0. The sample was cooled to room temperature after an agitation at 60°C for 1 h. The resulting black precipitate was centrifuged at 8000 rpm for 5 min and washed three times with ethanol (95%), finally dried in air naturally.

#### Immobilization of Rhamnosidase on Fe_3_O_4_@GO

The immobilization of the rhamnosidase was performed as follows: 5 mg Fe_3_O_4_@GO was dispersed in 4 mL citrate buffer (50 mM), pH = 6.0, and 1 mL rhamnosidase and 0.1% genipin were added and placed in a centrifuge tube under vibration in a thermostatic oscillator (DDHZ-300, Shanghai, China) with a speed of 170 rpm and kept at 30°C for 24 h. The immobilized enzyme was collected with a permanent magnet, then washed thoroughly with 50 mM citrate buffer pH = 6.0 and stored at 4°C until use.

#### Characterization

##### SEM

The morphologies of all dried GO and Fe_3_O_4_@GO were characterized by a scanning electron microscope (EVO18, ZEISS, Germany). The sample was sputtered with gold, the images of the coating sample were observed at 1000 times magnification under the acceleration voltage of 10.0 kV.

##### FT-IR spectra

In order to identify the possible structure difference between GO and Fe_3_O_4_@GO, the two samples for FT-IR measurement within the range 500–4000 cm^–1^ were prepared by mixing with potassium bromide (KBr), respectively, and then compressed into thin pellets (Vector33, Bruker, Germany) (Zhang et al., 2010).

##### XRD

The crystal morphology and the structure of GO and Fe_3_O_4_@GO powder were assessed with XRD analysis. The pattern of the sample was recorded on a Bruker polycrystalline X-ray diffraction (Cu-Kα radiation, λ = 1.5406 Å) at a scanning speed of 120/min in the 2θ range of 10–90°.

##### Thermal study

TG-DSC curves were observed with a Simultaneous Thermal Analyzer (STA449 F3, NETZSCH, Germany), 4 mg of samples were used, and a continuous heating ramp of 10 kmin^–1^ from 30°C to 500°C was adopted in a nitrogen atmosphere.

### Integration and Separation of Hesperidin and Its Coordination With Cu^2+^

The preparation of hesperidin copper complex has been slightly modified according to the method previously used by our research group (Gao et al., 2018). The hesperidin-loaded D296 resins were packed into an ion exchange column (22 mm × 480 mm). The eluent made of methanol solution containing copper chloride flowed out at a rate of 4 mL per minute and samples were collected using an automatic partial collector (40 mL per tube). And the sample with the highest absorbance at the specific absorption peak was concentrated in a rotary evaporator (RE-52AA, Shanghai, China) at 50°C. The concentrated solution was cooled to room temperature and dried with a freeze dryer (SCIENTZ-18N, Ningbo, China) for 24 h to obtain a brown and slightly yellow hesperidin-Cu (II) complex.

### Catalytic Experiments

#### Determination of the Enzyme Activity

An aliquot hesperidin-copper complex solution (10 mL, 0.1 mg/mL) was prepared with citric acid buffer 50 mM (pH 6.0) as the reaction substrate and preheated in an oscillator at 60°C for 5 min, followed by adding rhamnosidase to start the catalysis. After oscillating for 1 h (170 r/min), it was immediately removed and inactivated with boiling water for 30 min. The solution after enzyme elimination was centrifuged at 4000 r/min for 20 min, then the supernatant was passed through the 0.22 μm filter membrane. The content of hesperidin-Cu, HMG-Cu in the reaction solution was determined by HPLC. One enzyme unit (U) was considered the amount required for enzyme liberation of 1 μg HMG per minute under the described reaction conditions.

#### Determination of the Optimum pH

The activity of free and immobilized rhamnosidase (1.98 U) was assayed in buffers of varying pH 2.0–9.0. The buffers used were glycine-HCl (pH 2.0), sodium citrate (pH 3.0, 4.0, 5.0, and 6.0), sodium phosphate (pH 7.0 and 8.0) and Tris–HCl (pH 9.0). Measurements of enzyme activity were carried out under the conditions described above.

#### Determination of the Optimum Temperature

The activity of free and immobilized rhamnosidase was measured in 0.05 M sodium acetate buffer (pH 6.0) at different temperatures (20–80°C) under the same experimental methods as indicated before.

#### Thermal Stability

The thermal stability of the enzyme was assayed by heating the free and immobilized enzyme at the temperatures of 60°C in sodium citrate buffer solution (50 mM, pH 6.0) for altering times in the absence of substrate. Subsequently, the hesperidin-copper solution (as the substrate) was added and the measurement of the enzyme activity was determined as described above.

#### Determination of Kinetic Parameters

For determining the Michaelis–Menten constant (*K*_m_) and the maximum rate of the reaction (*V*_max_), an activity experiment was conducted by varying the concentration of the substrate (from 20 to 100 μg/mL). The double reciprocal curves of Lineweaver–Burk were plotted to calculate *K*_m_ and *V*_max_ values according to Michaelis-Menten equation (1) as follows:

(1) $$\frac{1}{V}=\left(\frac{K_{m}}{V_{m\,a\,x}}\times \frac{1}{\left[S\right]}\right)+\frac{1}{V_{m\,a\,x}}$$

where the slope was $\frac{K_{m}}{V_{m\,a\,x}}$, the intercept was $\frac{1}{V_{m\,a\,x}}$.

### Production and Validation of HMG

After the reaction on the immobilized rhamnosidase, the mixture was separated by a permanent magnet, thus the biocatalyst was removed, washed and reused. The analysis of products was performed by HPLC. Excess ammonium hydroxide as a dissociation agent was used and added to the supernatant, then stirred for 1 h and pH was adjusted to neutral, the mixture stood for 30 min. A white precipitate was obtained through centrifuging the turbid liquid, washed three times with distilled water and then dried in a freeze drier. UV-Vis spectroscopy was determined by a TU-1901 spectrophotometer (Beijing, China), the spectral scanning region of the target product (70% ethanol as solvent) was from 200 to 500 nm, and the differences of the spectra were compared before and after dissociation. In addition, the molecular weight of the target product was verified by mass spectrometry.

## Results and Discussion

### Characterization of Fe_3_O_4_@GO

#### SEM Analysis

**FIGURE 2:**
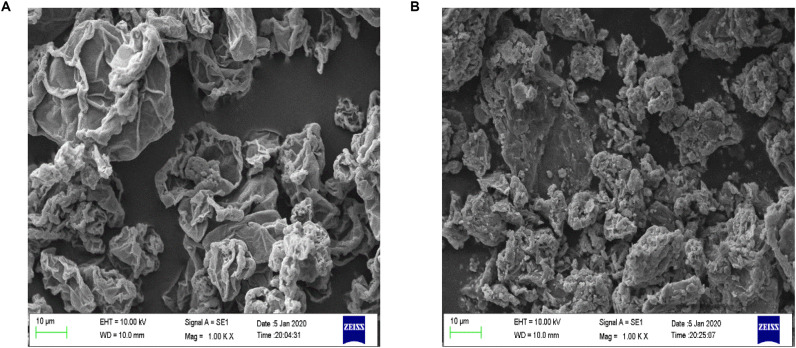
Surface morphology of GO **(A)** and Fe_3_O_4_@GO **(B)**.

The surface morphology of Fe_3_O_4_@GO and GO was observed by SEM under 1KX magnification, and the images were presented in Figure 2. The surface of GO had some wrinkles and the edges curled slightly (Patel et al., 2017). The Fe_3_O_4_ particles can be seen that it adhered and dispersed on the surface of GO.

#### FT-IR Analysis

**FIGURE 3:**
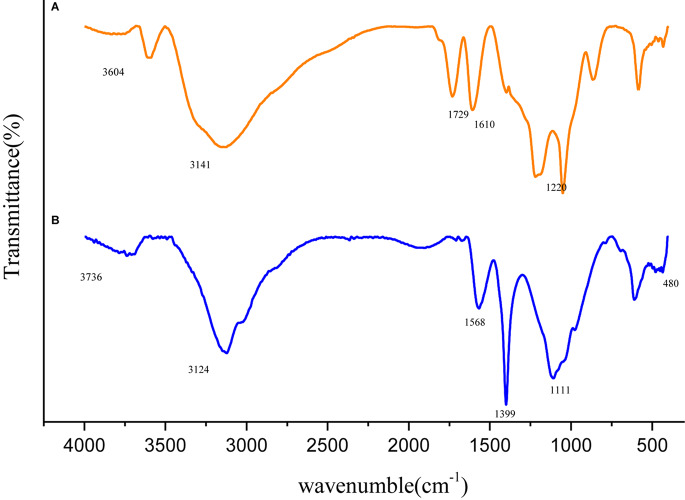
Infrared spectra of GO **(A)** and Fe_3_O_4_@GO **(B)**.

Variation in molecular structure was determined by infrared spectroscopy and the spectra of GO and Fe_3_O_4_@GO was shown in Figure 3. In the spectrum of GO, the peak appeared at 1729 cm^–1^ attributed to the stretching band of C = O in carboxylic acid or carbonyl moieties, the rest peaks of 3604, 1399, 1220 cm^–1^ could be corresponding to the stretching vibration of O-H, C-O, O-H groups (Kassaee et al., 2011). The Fe_3_O_4_@GO spectrum differed in that the peak of O-H at 3736 cm^–1^ was obviously weaken, and the Fe-O stretching peak approximately at 480 cm^–1^ could be seen, which confirmed Fe_3_O_4_ particles successfully anchored onto the GO (Fan et al., 2017). In addition, the Fe_3_O_4_@GO curve was a little blue-shift relative to the GO curve, the reason may be that the large Fe_3_O_4_ particle loaded on the surface of GO, leading to a change in the charge density (Peng-Fei et al., 2000).

#### XRD Analysis

**FIGURE 4:**
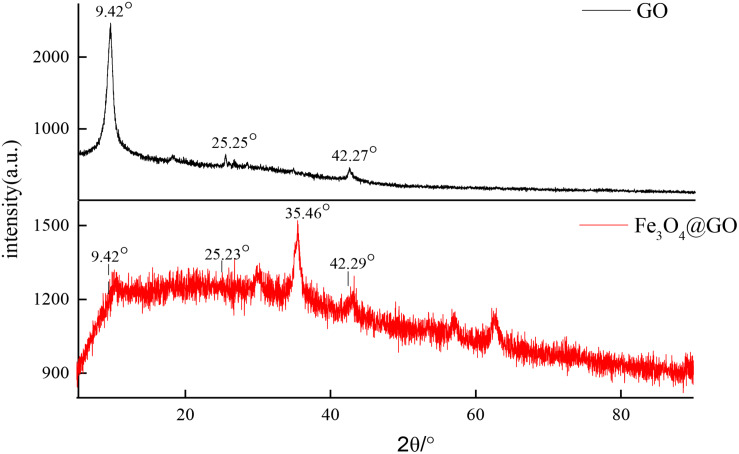
X-ray diffraction patterns of GO and Fe_3_O_4_@GO.

In order to investigate the effect of Fe_3_O_4_ on molecular crystal structure, X-ray diffraction patterns of GO and Fe_3_O_4_@GO powders were determined as shown in Figure 4. The diffraction peaks of GO were obviously different from that of the combined Fe_3_O_4_@GO composite material. GO curve demonstrated well distinguished characteristic diffraction peaks at the 2θ values 9.42, 25.25, 42.27°, respectively. But a portion of Fe_3_O_4_@GO scattering from the crystalline domains was diffuse and displayed poor crystalline feature compared with GO (Liu et al., 2010; Mondal et al., 2014), only a slightly strong diffraction peak appeared at 35.46°, perhaps it was the introduction of Fe_3_O_4_ that caused the change in crystal morphology. The three diffraction peaks in GO can also be found in Fe_3_O_4_@GO pattern, may indicate that the basic skeleton of GO had not changed.

#### TG-DSC Analysis

**FIGURE 5:**
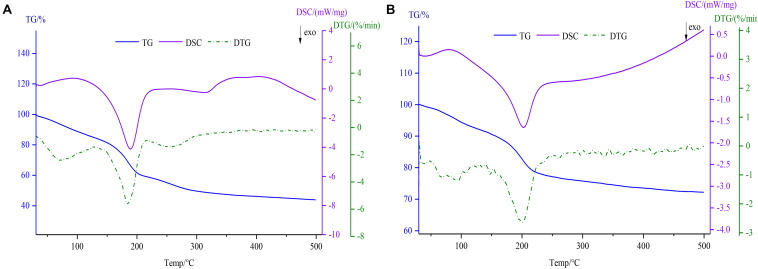
TG-DSC-DTG curves of GO **(A)** and Fe_3_O_4_@GO **(B)**.

As shown in Figure 5, thermodynamics data including TG, DSC, and DTG was determined using a simultaneous thermal analyzer. In the TG and DTG thermograms, the degradation of GO (Figure 5A) was divided roughly into three steps (de Jesus et al., 2019). Firstly, the weight loss (14.96%) due to free water evaporation was observed at 31–126°C for GO. The mass loss of the second stage within the temperature range of 126–213°C was 25.64%, the reason for the mass loss may be related to the loss of the combined water. In the last process, the residual weight remained 43.8% after increasing the temperature till 500°C. However, the Fe_3_O_4_@GO (Figure 5B) powders showed a two-step weight decrease (7.76, 19.96%) because of free water evaporation within the temperature range of 30–127°C and Fe_3_O_4_@GO combustion at temperatures between 127 and 500°C (remained 72.38%), which was similar to the literature (Patel et al., 2017). As can be seen from the DTG curves, the temperature corresponding to the peak of Fe_3_O_4_@GO was slightly higher and since its mass loss was obviously smaller than that of GO, it could be concluded that the thermal stability improved to some extent after being combined with iron. The DSC curves showed two exothermic peaks of GO and Fe_3_O_4_@GO at 189.1, 203.1°C, respectively, which were in accord with the second phase of weight loss by TG (Mohan et al., 2019). Due to the evaporation of combined water, which was an endothermic process as depicted by the endothermic peak of DSC curve (Yi et al., 2017).

#### The Magnetic Property

**FIGURE 6:**
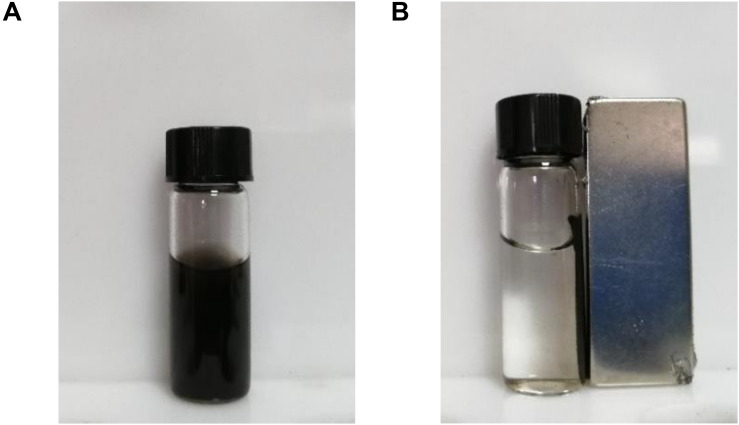
Images of GO **(A)** and Fe_3_O_4_@GO **(B)**.

Graphene oxide and Fe_3_O_4_@GO of the same mass were diffused in distilled water, and it was found that GO was evenly dispersed, while Fe_3_O_4_@GO was precipitated after a period of time. Through the water retention experiment, it was found that the value decrease of Fe_3_O_4_@GO compared to GO may be due to the presence of adsorbed iron. The low water retention capacity of Fe_3_O_4_-loaded GO may improve physical stability (Altundogan, 2005). When placing a permanent magnet on the side, the magnetic particles were effectively separated in a short time, which could be seen from Figure 6 (Hua et al., 2014). Perhaps the magnetic property benefits to the following separation of hydrolytes with immobilized enzyme on Fe_3_O_4_@GO.

#### Interaction Between Hesperidin-Cu (II) Complex and Immobilized Enzyme

The hesperidin-Cu (II) complex (Figure 1) was obtained by the method of separation reaction integration mentioned above. And the formation of the complex was confirmed and the carbonyl group and the hydroxyl group in hesperidin were involved in the coordination reaction in our former research (Chen and Zhu, 2018). Due to the “concentration effect” or the “embedding effect” of rutinose on the ligand: coordination caused copper ion buried in the water-insoluble glycoside ligand (hesperetin) in the middle of the complex molecule (Yu et al., 2007). As a result, copper ions had no space to interact with Fe_3_O_4_@GO, while the rutinose part of hesperidin reacted with glycosidase, so the copper would not affect the enzyme and immobilized enzyme materials.

### Effects of Reaction pH and Temperature on Enzyme Activity

**FIGURE 7:**
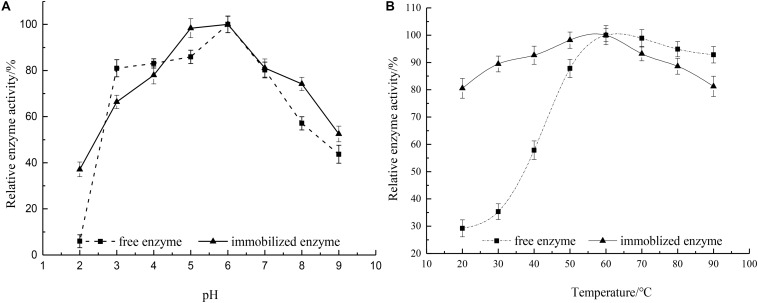
**(A)** Influence of pH on relative activity of free and immobilized rhamnosidase (—■—) free enzyme and (**—**▲**—**) immobilized enzyme. **(B)** Influence of temperature on relative activity of free and immobilized rhamnosidase (—■—) free enzyme and (**—**▲**—**) immobilized enzyme.

The water solubility difference between the two was measured by the standard curve method, and it was found that the complex of the same quality was 3.36 times more soluble than that of hesperidin, thus the effect of pH on enzyme activity was determined using hesperidin-Cu(II) as the substrate within a pH region of 2.0 to 9.0. Figure 7A demonstrated the pH-activity profiles for free and immobilized rhamnosidase. The optimum pH of the immobilized enzyme was 6.0, the same as that of the free enzyme. The influence of the microenvironment around the active site of enzyme protein on the hydrogen-ion concentration could be observed by comparing the pH of the two. And the result demonstrated that the support hardly altered the proton distribution between the aqueous phase and the microenvironment surrounding the active site of the enzyme (Busto et al., 2007). The immobilized enzyme presented better tolerance of low pH than the free rhamnosidase. The activity of the free enzyme at pH 2.0 remained only 5.96%, while the other still maintained 43.7% activity under the same experimental conditions (Khan et al., 2017).

The relative activity of the free and immobilized rhamnosidase as a function of temperature was shown in Figure 7B. The optimal temperature for free and immobilized enzymes was noticed to be same at 60°C. The trend of the curve indicated that the free enzyme was easily affected by the temperature, but the temperature variation had less effect on the immobilized enzyme. Because the immobilization reduced the activation energy of the enzyme, leading to the improvement of the catalytic efficiency for the immobilized rhamnosidase (Soares and Hotchkiss, 1998). In the range of 70–90°C the free enzyme retained at least 90% activity, therefore, showed good resistance to high temperatures.

### Thermal Denaturation

**FIGURE 8:**
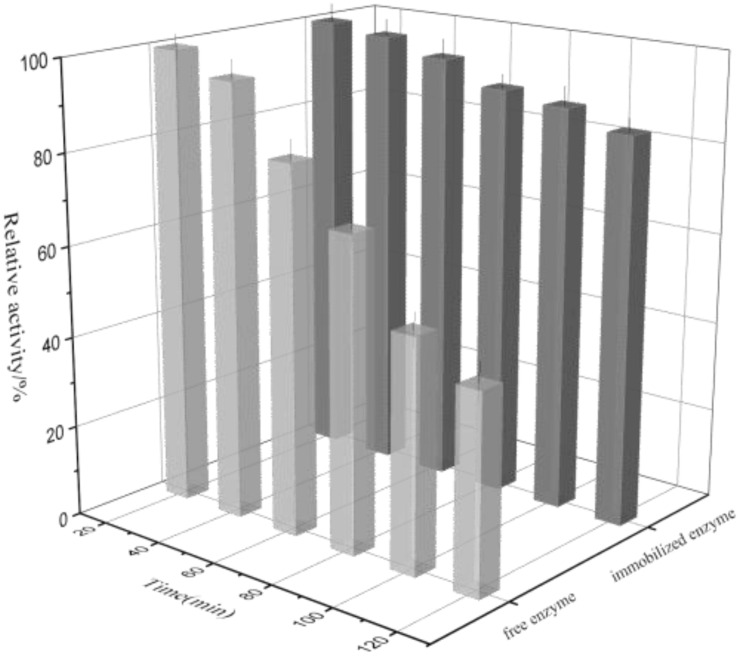
Thermal denaturation of free and immobilized rhamnosidase.

The thermal denaturation plots for free and immobilized enzyme were depicted in Figure 8. It can be seen that the free enzyme exhibited 43.4% activity at the time of 120 min while the immobilized rhamnosidase retained 85.3% activity in the similar experimental conditions. The reason may be that the immobilized matrix endowed with the enzyme rigidity again, making it more resistant to thermal denaturation.

### Kinetic Parameters Investigation

The kinetic parameters (*K*_m_ and *V*_max_) of rhamnosidase in free and immobilized forms were summarized in Table 1. *K*_m_ value is a characteristic value independent of substrate and enzyme concentration, which is often used to evaluate the ability of enzymes and substrates to form complexes. As can be seen from the current data, the *K*_m_ value of immobilized enzyme was higher than that of free enzyme, indicating that both free and immobilized enzymes had high affinity to the substrate (Yang et al., 2019). *V*_max_ represents the maximum reaction rate under a certain amount of enzyme, that is, the reaction rate when the enzyme is completely saturated with substrate, proportional to the enzyme concentration. *V*_max_ of the immobilized enzyme (0.7612) was slightly higher than that with free enzyme (0.7467), certifying Fe_3_O_4_@GO was a good carrier while maintaining the enzyme activity.

**TABLE 1:** Kinetic parameters of free and immobilized rhamnosidase.

**Sample**	***K*_m_/(μg⋅mL^–1^)**	***V*_max_/μg/(mL⋅min)**
Free enzyme	479.0426	0.7467
Immobilized enzyme	489.6313	0.7612

### Reusability Assay

**FIGURE 9:**
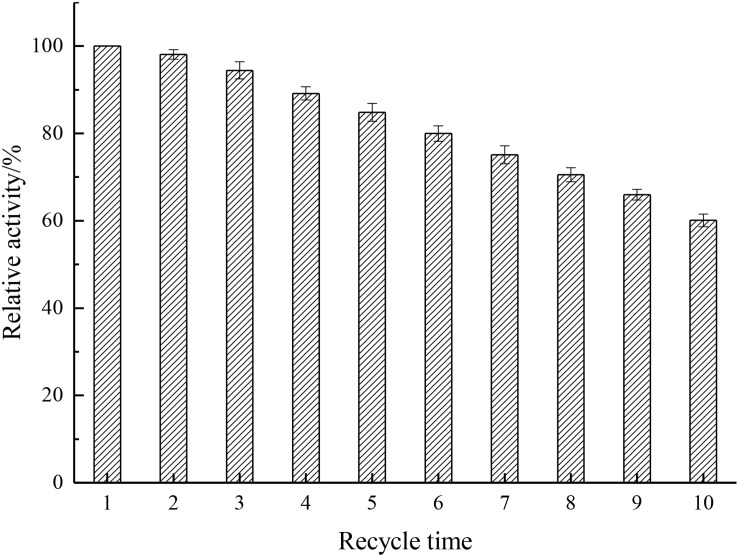
Reusability of the immobilized rhamnosidase.

Analysis of the reusability can provide cost-effectiveness for the use of enzymes in economically viable catalytic processes (Haider and Husain, 2007). As depicted in Figure 9, results showed that the relative activity decreased by only 1.89% after the first cycle and kept 60.1% after 10 cycles, indicating that the recycling stability of immobilized enzyme was enhanced by the introduction of Fe_3_O_4_@GO, which endowed the enzyme better rigidity.

### Production of HMG

**FIGURE 10:**
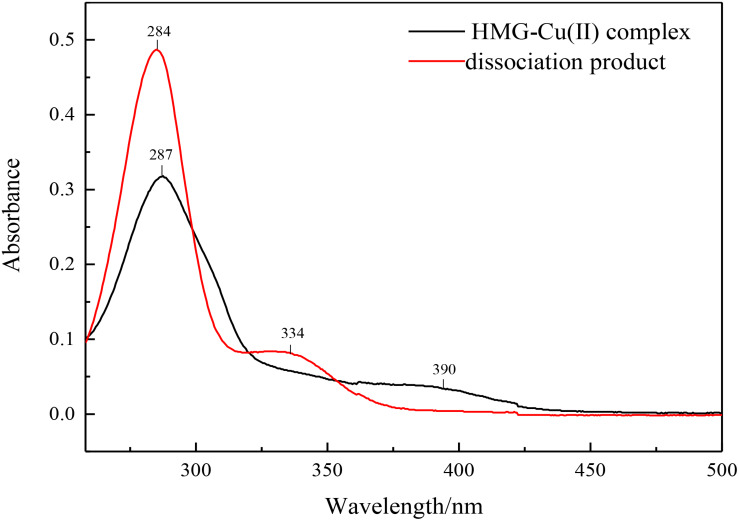
UV–Vis spectra of HMG and ligand-dissociation product.

The enzymatic hydrolysate was separated from the immobilized enzyme by a permanent magnet, and the product conversion was measured by HPLC. Excess ammonium hydroxide as a dissociation agent was used and added to the supernatant to free target product, the obtained precipitate was washed and dried, characterized by UV-Vis spectroscopy. The characteristic absorption peaks at 390 and 287 nm had a blue shift to 334 and 284 nm, which was corresponding to the HMG spectra and indicated the success of the solution dissociation (Figure 10).

### Mass Spectra

**FIGURE 11:**
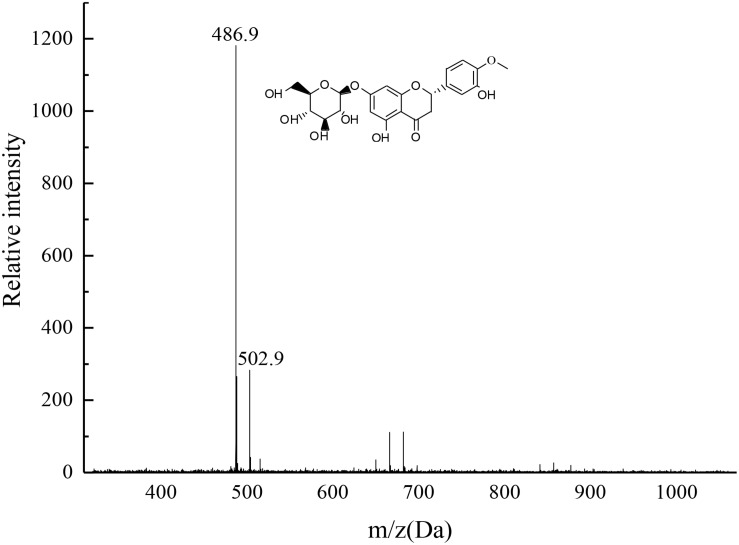
TOF-MS spectra of the target product HMG.

The target product was dissolved by chromatographic methanol and the TOF mass spectra was plotted as shown in Figure 11. The two most intense peaks were observed at m/z 486.9 and at m/z 502.9, confirming the product was consistent with HMG. The 486.9 Da peak was explained by the composition of a unit plus, i.e., [M + Na^+^]^–^, whereas the peak of 502.9 Da was due to the presence of K^+^, suggesting the possible molecular formula of M was C_22_H_24_O_11_ (the possible structure as depicted in Figure 11). The reason might be that the target sample contained oxygen on the carbonyl group, which was the action site for easy binding with Na^+^ or K^+^. The residual two ions in the mobile phase, the system pipeline and other places could easily combine with the compound and form sodium-containing or potassium-containing fragment peaks (Pasch et al., 2001).

## Conclusion

Hesperetin-7-O-glucoside, a kind of highly bioactive citrus flavonoids, has been successfully created by constructing a high-effective and green biosynthesis pathway. The green pathway integrates following steps like the construction of Fe_3_O_4_@GO as a matrix, the immobilization of the rhamnosidase on the matrix as a biosynthesis platform, the enzyme hydrolysis using a novel substrate of hesperidin-Cu (II) and the immobilization enzyme, the magnetic separation of hydrolytes with the enzyme, and the ligand dissociation of HMG-Cu(II) using ammonium water. Ionic liquid can be used as a new solvent or cosolvent for organic synthesis or biocatalytic transformation (Lou et al., 2006). Considering the problem of saving technological process, in the future research, we will explore whether ionic liquid can improve the solubility of hesperidin, so as to directly act as a substrate reaction and avoid the steps of complexation and dissociation.

Firstly, the magnetic Fe_3_O_4_@GO was prepared as a matrix material, easily realizing the objectives of high immobilization efficiency, feasible enzyme recycling, and good separation of hydrates with the immobilized rhamnosidase. In addition, genipin was used as a green cross-linker in the immobilization process. The characterization was used to determine the difference before and after combining Fe_3_O_4_, including FTIR, XRD, SEM and thermal analysis. Results indicated that Fe_3_O_4_@GO could be an ideal carrier for enzyme immobilization based on its effective dispersing properties and structure with good rigidity.

Secondly, by comparing the effect of pH and temperature on immobilized enzyme and free enzyme, the immobilized rhamnosidase was less affected by temperature and pH during the reaction process, and its thermal stability was improved in comparison with that of free rhamnosidase. Furthermore, the kinetic values (*K*_m_ and *V*_max_) of immobilized enzyme calculated a little higher than the free enzymes, confirming that the affinity between enzyme and substrate was improved by immobilization. The immobilized rhamnosidase also showed slightly better activity after successive utilization of 10 runs. In addition, the immobilization enzyme can easily be separated with hydrolytes through the attraction of a permanent magnet.

Thirdly, by introducing ligand dissociation agent like ammonium water competing for combination with metal ions, the obtained object enzymatic hydrolysate of HMG was separated from copper and precipitated. The ultraviolet spectrum indicated that the ligand dissociation matter was successfully created, and mass spectrometry was performed to verify that the product obtained was the target material HMG. Table 2 listed the differences between some references’ results and this study, proving the novelty of the reaction pathways and materials used in the paper.

**TABLE 2:** The methods differences between literatures.

**Methods**	**Literatures**	**Differences**
The preparation of metal-hesperidin analogs complexes	Gao et al. (2018)	The product was prepared by an integrated separation reaction pathway, but the substrate was not a derivative of hesperidin.
	Chen and Zhu (2018)	The product purity of zinc-hesperidin complex prepared by solution coordination was lower than that prepared by separation and integration method.
	Carceller et al. (2019)	In this paper, GO was used to immobilize rhamnosidase for selective synthesis of citrus flavonoids prunin and naringenin, but no magnetic separation.
Biosynthesis pathway of hesperetin analogs	Koseki et al. (2008)	The study focused on the hydrolysis ability of extracted rhamnosidase to flavonoids and did not immobilize the enzyme.
	Yadav et al. (2012)	The enzymatic properties of α-L-rhamnosidase were studied after it was purified without immobilization, and hydrolyzed naringin, rutin and tangerine to liberate L-rhamnose.
	Patel et al. (2017)	Reduced GO-Fe_3_O_4_ was synthesized to support enzyme immobilization. Unlike the enzyme immobilized in my paper, and the immobilized material GO was reduced.
The immobilization by magnetic Fe_3_O_4_@GO	Fan et al. (2017)	A composite material (MIP@Fe_3_O_4_@GO) was used to simultaneously separate and enrich two alkaloids (evodiamine and rutaecarpine) in the extract of evodiae fructus.
	Hua et al. (2014)	The oxidative degradation characteristics of bisphenol A in a heterogeneous Fenton reaction catalyzed by Fe_3_O_4_/GO were studied. It is different from the application of the composite in my paper.

So, the constructed pathway is successful because it is based on hesperidin hydrolysis using a immobilization rhamnosidase on Fe_3_O_4_@GO as a biosynthesis platform with good rigidity, magnetic separation property and reusability, and it is also based on the good ligand solubility using hesperidin-Cu (II) as enzyme substrate and good ligand dissociation property using ammonium water as a ligand dissociation agent. In addition, the raised biocatalyst pathway based on the biosynthesis platform, may possess a great sustainable and green application prospect by creating HMG or other flavonoids on a large scale.

## Data Availability Statement

The datasets generated for this study are available on request to the corresponding author.

## Author Contributions

SZ designed and implemented the research. WW and YG performed the experiments. NX supervised the whole process. WW and QL sorted and analyzed the data. WW and NX wrote the manuscript. All authors contributed in the manuscript revision.

## Conflict of Interest

The authors declare that the research was conducted in the absence of any commercial or financial relationships that could be construed as a potential conflict of interest.
